# Impact of Muscle Mass on the Performance of Creatinine‐Based eGFR Equations and Mortality Risk Assessment After Kidney Transplantation

**DOI:** 10.1002/jcsm.70032

**Published:** 2025-09-29

**Authors:** François Gaillard, Melissa Ould Rabah, Olivier Aubert, Nicolas Garcelon, Antoine Neuraz, Christophe Legendre, Dany Anglicheau, Dominique Prié, Frank Bienaimé

**Affiliations:** ^1^ Service de Transplantation, Néphrologie et Immunologie Clinique Hôpital Edouard Herriot et Faculté de Médecine, Université Lyon 1 Lyon France; ^2^ Research on Healthcare Performance RESHAPE INSERM U1290, Université Claude Bernard Lyon 1 Lyon France; ^3^ Service de Physiologie Hôpital Necker‐Enfants Malades, Assistance‐Publique‐Hôpitaux de Paris Paris France; ^4^ Faculté de médecine Université de Paris‐Cité Paris France; ^5^ Service de Néphrologie et Transplantation Hôpital Necker‐Enfants Malades, Assistance‐Publique‐Hôpitaux de Paris Paris France; ^6^ Institut Necker‐Enfants Malades, INSERMU1151 Paris Paris France; ^7^ Université de Paris, Imagine Institute, Data Science Platform, INSERM UMR 1163 Paris France; ^8^ Service d'Informatique Médical Hôpital Necker‐Enfants Malades, Assistance‐Publique‐Hôpitaux de Paris Paris France

**Keywords:** creatinine, kidney transplantation; estimated GFR, measured GFR, mortality, muscle mass

## Abstract

**Background:**

Estimating glomerular filtration rate (eGFR) in kidney transplant recipients (KTR) typically relies on plasma creatinine, which is influenced by muscle mass. Reduced muscle mass is suspected to reduce eGFR performance in this population but this effect has not been rigorously evaluated. This study quantified the impact of muscle mass on eGFR accuracy and its confounding effect on the association between kidney function and mortality in KTR.

**Methods:**

We studied a prospective and consecutive cohort of 1829 KTR (mean age 52 ± 14 years; 38.9% female) who underwent GFR measurement using iohexol clearance (ioGFR). Muscle mass was assessed by creatinine excretion rate (CER) from timed urine collections. We evaluated the impact of muscle mass on the performance of five eGFR equations (MDRD, CKDEPI_2009_, CKDEPI_2021_, EKFC and RFKTS) using multiple regression and subgroup analysis. The association between eGFRs, ioGFR and mortality was examined using Cox proportional hazards models.

**Results:**

All eGFR equations showed a significant negative correlation with CER. EKFC was the least sensitive to CER (***β*** coefficient 95% confidence interval [CI]: −0.17 to −0.12). All eGFR equations demonstrated reduced accuracy in the lowest muscle mass tertile. In multivariable analyses, ioGFR was significantly associated with mortality (hazard ratio 95% CI: 0.972–0.995) but eGFRs were not. Including CER in the Cox models resulted in convergence of the mortality hazard ratios for ioGFR and eGFRs (hazard ratio 95% CI: ioGFR: 0.98–0.999; MDRD: 0.98–0.999; CKDEPI_2021_: 0.99–1; EKFC (0.98–1) RFKS: 0.98–0999).

**Conclusion:**

The performance of all tested creatinine‐based eGFR equations is strongly impacted by muscle mass. Muscle mass is also a key confounder in the mortality risk assessment using eGFR. Incorporating muscle mass into KTR's evaluations may improve kidney function assessments in KTR.

**Abbreviations**
BMI: body mass index
CER: creatinine excretion rate
CI: confidence interval
eGFR: estimated glomerular filtration rate
eGFRcr: creatinine‐based eGFR
GFR: glomerular filtration rate
HR: hazard ratio
ioGFR: iohexol plasma clearance
KT: kidney transplantation
KTR: kidney transplant recipients
PCr: urinary protein to creatinine ratio

## 1 Introduction

The adequate care of kidney transplant recipients (KTR) requires frequent monitoring of glomerular filtration rate (GFR), in particular in the first months/years following transplantation. Monitoring of GFR in KTR relies on GFR estimation (eGFR) from endogenous markers, mainly plasma creatinine [1]. This strategy based on simple plasma markers is far more convenient and available than GFR measurement from exogenous tracers such as iohexol, iothalamate or 99mTc‐DTPA. Creatinine is almost exclusively produced by the muscle then eliminated by the kidney, mainly through glomerular filtration with a small contribution of secretion by the tubule. In stable conditions, the creatinine plasma value is the one allowing an equilibrium state between creatinine production and elimination. Therefore, muscle mass and GFR are the main determinants of plasma creatinine level. Consequently, creatinine‐based eGFR (eGFRcr) equations can be considered as implicit estimators of muscle mass, which is derived from only two parameters: gender and age. Such an approach is not fully satisfying, especially in populations whose muscle mass distribution diverges from the population that was used to develop the equation. Consequently, eGFRcr will overestimate the GFR of patients with an unusually low muscle mass for a given gender and age [2]. Importantly, a low muscle mass is also a strong predictor of mortality in multiple populations, including KTR [3, 4, 5, 6, 7, 8, 9, 10, 11]. Thus, muscle mass represents a potential confounder in studies assessing the link between kidney functions and mortality. Indeed, a recent study of a large cohort of non‐transplanted patients demonstrated that low muscle (evaluated by 24 h urinary creatinine) mass was the critical factor explaining the paradoxical increase in mortality associated with a high eGFRcr [12].

The distribution of muscle mass in KTR may plausibly diverge from the one observed in non‐transplanted patients suffering from chronic kidney disease. Several factors affect KTR muscle mass, such as the time spent on dialysis, surgery or corticosteroids [13]. These factors do not apply homogeneously to each KTR and their intermeshing should predictably increase muscle mass dispersion in a manner that is not necessarily related to age or gender. This process is further dynamic: kidney transplantation is associated with a loss of muscle mass as early as 1 month, with incomplete correction at 1 year [14].

It is usually stated that variations in muscle mass contribute to a decrease of eGFRcr accuracy in KTR [1]. Yet, the impact of muscle mass on eGFRcr has also been proposed to confound its association with mortality [15]. A comprehensive analysis of the effect of muscle mass on these parameters is lacking to date.

In this context, we studied 1829 KTR with measured GFR by iohexol plasma clearance (ioGFR) and muscle mass evaluation by in‐hospital measurement of creatinine excretion rate (CER) with multiple timed collection (a method that we previously validated against bio‐impedance spectrometry [8]) to assess:

1. The contribution of muscle mass to eGFRcr and its effect on equations accuracy.
2. how muscle mass confounds the association between eGFRcr and mortality.

We tested 4 creatinine‐based equations developed in cohorts that did not include KTR: (MDRD [16], CKDEPI_2009_ [17], CKDEPI_2021_ [18] and EKFC [19]) and the RFKTS (Race Free Kidney Transplant Specific eGFR; [22]), which was specifically developed and validated in KTR cohorts.

## 2 Material and Methods

### 2.1 Study Population

**FIGURE 1:**
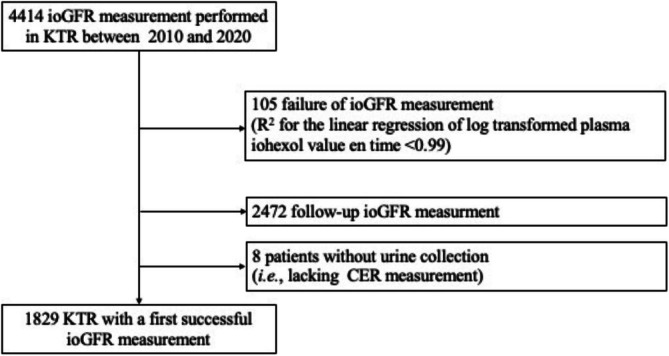
Study flow chart.

All patients were adults transplanted and followed in the department of renal transplantation at Necker Hospital, Paris, France. According to our standard follow‐up protocol, patients with a stable allograft function undergo GFR measurement 3 months, 1 year and then every other year after transplantation. According to French law, anonymous retrospective data do not require authorization from an institutional review board [20]. All the patients with a successful first mGFR measurement by iohexol plasma clearance from November 2011 (when creatinine measurement started to be performed with an IDMS traceable method) to March 2020 were included in the study (Figure 1).

### 2.2 Data Collection

The prospective database, Données Informatiques Validées en Transplantation (DIVAT clinical prospective cohort, official website: www.divat.fr; registration number: 1016618) was used to collect data at specific points for each patient (3 months at follow‐up). The data include information concerning the donor (age, sex, deceased or living donor, cold ischemia time), the recipient (age, sex, primary cause of kidney disease), the transplantation procedure, the immunosuppression regimen, the occurrence of delayed graft function (defined as the need for haemodialysis during the first week after transplantation) and the occurrence of death or graft loss as defined by re‐transplantation or a return to long‐term dialysis. Death or allograft loss was reported to the research assistant implementing the DIVAT database. We prospectively collected height, body weight, BP and standard biochemical parameters at the time of GFR measurement. Patients were censored at the date of last recorded follow‐up. As follow‐up was conducted entirely within our centre, loss to follow‐up was minimal and most patients were censored because they remained event‐free at the end of the study period.

### 2.3 GFR Determination From Iohexol Plasma Disappearance Curves

Measured GFR (ioGFR) was calculated from the plasma disappearance curve of iohexol with hourly plasma samples from 2 to 5 h after iohexol injection using Jens Bröchner‐Mortensen's quadratic correction as previously described [21].

### 2.4 Laboratory Assays

Urinary and plasma creatinine measurement is routinely and stably performed with an IDMS traceable enzymatic assay (Multigent; Abbott) since February 2011 in our institution. CER was determined as the mean of the values obtained on successive urine samples collected every hour for 5 h. We previously reported the correlation between mean urinary CER and lean tissue mass [8].

### 2.5 Equations to Estimate GFR From Plasma Creatinine

We evaluated five equations as previously reported: the modified diet in renal disease equation (MDRD) [16], the 2009 chronic kidney disease epidemiology equation (CKDEPI_2009_) [17], the 2021 chronic kidney disease epidemiology equation (CKDEPI_2021_) [18], the European kidney function consortium (EKFC) [19] equation and the race‐free kidney transplant‐specific equation (RFKTS) [22].

### 2.6 Statistical Analyses

Data were analysed using R version 4.5.0. (R Core Team (2020). R: A language and environment for statistical computing. R Foundation for Statistical Computing, Vienna, Austria. URL https://www.R‐project.org/). Data are expressed as mean ± SD for normally distributed continuous variables and as median (25th–75th percentiles) for continuous variables with a skewed distribution. Qualitative variables are reported as absolute numbers and percentages.

We evaluated the performance of five equations to estimate GFR in our population. We calculated the correlation, the Lin concordance correlation coefficient, the percentage of eGFR within 30% of ioGFR value and the percentage of eGFR within 10% of ioGFR value.

We calculated the hazard ratio (HR) for death using CER as a continuous variable in a univariate Cox model. We designed multivariate Cox proportional hazards models for death. We included a maximum of 15 prespecified explanatory covariates in the Cox models, which were selected on the basis of their reported association with the outcome in other studies: ioGFR, gender, smoking, coronary artery disease, preemptive transplantation, donor age, donor type, diabetes, recipient age, systolic blood pressure, height, weight. Missing data were omitted from the Cox models. The proportionality was tested for each covariate based on weighted residuals. All clinical and biologic parameters included in the multivariable models were measured/assessed at the inclusion of the patients in the study. We generated a correlation matrix to assess potential multicollinearity across variables (Figure S1). ioGFR was not strongly correlated with other covariates.

## 3 Results

### 3.1 Characteristics of the Population

The cohort consisted of 1829 patients who were transplanted at Necker hospital between June 2005 and March 2020 and who were addressed to the physiology department for GFR measurement. Characteristics of patients are summarized in Table 1.

**TABLE 1:** Characteristics of patients included in the study.

Parameter	Values	Total (*n* = 1829)
Age	Median [Q25–Q75]	52 ± 14
Gender (Female)	*N* (%)	703 (38.5)
Initial nephropathy	*N* (%)	
Cystic kidney disease		339 (18.5)
Glomerulonephritis		275 (15)
Diabetic nephropathy		146 (8)
Focal segmental glomerulosclerosis		124 (6.8)
Vascular nephropathy		113 (6.2)
Uropathy		110 (6)
CAKUT		75 (4.1)
Lupus nephritis		42 (2.3)
Genetic glomerular disease		37 (2)
Toxic nephropathy		30 (1.6)
Hemolytic and uremic syndrome		28 (1.5)
Unknown		368 (20.1)
Other		143 (7.8)
First transplantation		1529 (83.7)
Preemptive transplantation	*N* (%)	335 (18.3)
Active smoking	*N* (%)	264 (14.4)
Diabetes	*N* (%)	279 (15.3)
Coronary disease	*N* (%)	217 (11.9)
Deceased donor	*N* (%)	1341 (73.3)
Donor age (years)	Mean ± SD	54 ± 16
	Missing values	6
Cold ischemia time (min)	Median [Q25–Q75]	945 [180–1380]
	Missing values	40
Delayed graft function	*N* (%)	283 (15.5)
Systolic blood pressure (mmHg)	Mean ± SD	131.1 ± 15.2
	Missing values	16
Diastolic blood pressure (mmHg)	Mean ± SD	75 ± 11
	Missing values	17
Height (cm)	Mean ± SD	169.2 ± 9.7
Weight (kg)	Mean ± SD	72.2 ± 15
BMI (kg/m^2^)	Mean ± SD	25.2 ± 4.6
ioGFR (mL/min/1.73m^2^)	Median [Q25–Q75]	51.8 [41.8–62.5]
Plasma creatinine (μmol/L)	Median [Q25–Q75]	123 [99–153]
Proteinuria/Creatininuria (mg/mmol)	Median [Q25–Q75]	18.9 [11.7–32]
	Missing values	322
CER (μmol/min)	Median [Q25–Q75]	7.71 [6.10–9.59]
eGFR‐MDRD (mL/min/1.73m^2^)	Median [Q25–Q75]	48.5 [37.8–61.3]
eGFR‐CKDEPI_2009_ (mL/min/1.73m^2^)	Median [Q25–Q75]	52 [39.5–66.9]
eGFR‐CKDEPI_2021_ (mL/min/1.73m^2^)	Median [Q25–Q75]	55 [42–70.6]
eGFR‐EKFC (mL/min/1.73m^2^)	Median [Q25–Q75]	51.8 [39.8–65.8]
eGFR‐RFKS (mL/min/1.73m^2^)	Median [Q25–Q75]	52.7 [43.3–63.3]
Transplantation vintage (months)	Median [Q25–Q75]	3.2 [2.8–35]

Abbreviations: BMI, body mass index; CAKUT, congenital anomalies of the kidney and urinary tract; CER, creatinine excretion rate; eGFR, estimated glomerular filtration rate; ioGFR, GFR measured with iohexol plasma clearance; PCr, protein to creatinine ratio.

### 3.2 Performance of the eGFRcr Equations

We compared the performance of the 5 eGFRcr equations in our cohort. The correlation between eGFRcr and ioGFR was similar for the five equations, as was the Lin concordance correlation coefficient. The proportion of eGFR within 30% of ioGFR was lower for CKDEPI_2021_ as compared with all other equations. The proportion of eGFR within 10% of ioGFR was not significantly different between equations. These results are summarized in Table 2.

**TABLE 2:** Performance of the 4 equations to estimate GFR.

		MDRD	CKDEPI_2009_	CKDEPI_2021_	EKFC	RFKTS
R‐squared	CC (95% CI)	0.78 (0.76–0.80)	0.80 (0.78–0.81)	0.80 (0.78–0.81)	0.80 (0.78–0.82)	0.80 (0.78–0.82)
Lin CCC	CC (95% CI)	0.76 (0.74–0.78)	0.75 (0.73–0.77)	0.73 (0.71–0.75)	0.78 (0.76–0.79)	0.79 (0.78–0.81)
eGFR‐ioGFR (mL/min/1.73m^2^)	Median [Q25‐Q75]	‐2 [−8.4, 5.4]	0.4 [−6.3, 9.0]	3.1 [−3.8, 12.3]	0.3 [−6.3, 7.7]	1.2 [−4.8, 7]
(eGFR‐ioGFR)/ioGFR (%)	Median [Q25‐Q75]	‐4 [−16.8, 11.0]	0.8 [−13.2, 17.4]	6.4 [−8.1, 24.1]	7.1 [−1.4, 12.4]	2.2 [−8, 14]
Percentage within 30% of ioGFR	% (95% CI)	84.6 (82.9–86.2)	82.4 (80.6–84.0)	78.8 (76.8–80.6)	84.3 (82.5–85.9)	88.8 (87.2–90)
Percentage within 10% of ioGFR	% (95% CI)	35.3 (33.1–37.5)	35.1 (32.9–37.3)	32.9 (30.7–35.1)	36 (33.8–38.3)	42 (41–45)

Abbreviations: CC (95% CI), correlation coefficient (95% confidence interval); ioGFR, GFR measured with iohexol; Lin CCC, Lin concordance correlation coefficient.

### 3.3 Residual Impact of Muscle Mass on eGFRcr Estimations

To assess the quantitative contribution of muscle mass to eGFRcr across the five equations, we performed multiple linear regression with eGFR as the dependent variable and ioGFRcr and CER scaled to body size as explanatory variables (Table 3). We found significant negative correlations between CER and eGFRcr for all equations, with *β* coefficient ranging from −0.2 [−0.23 to −0.17] for MDRD to −0.14 [−0.17 to −0.12] for EKFC. The *R* ^2^ of the regression ranged from 0.68 (RFKTS) to 0.65 (MDRD) indicating that a non‐neglectable part of eGFRcr variation was neither explained by ioGFR nor CER. In complementary analyses, we divided the cohort according to gender‐specific CER/Height^2^ tertiles and assessed eGFRcr accuracy in these subpopulations (Table 4). For all equations, the absolute bias was higher in the low and high than in the central tertile of CER/Height^2^. In these extreme tertiles, grossly a quarter of the patients displayed an absolute difference between ioGFR and eGFRcr of more than 10 mL/min/1.73m^2^. Patients in the lowest tertile of muscle mass also displayed a lower ioGFR. Consequently, the accuracy (P30 and P10) of all eGFRcr equations was reduced in this tertile.

**TABLE 3:** Multivariable modelling of eGFRs according to ioGFR and CER.

		MDRD	CKDEPI_2009_	CKDEPI_2021_	EKFC	RFKTS
*β* (95% CI)	ioGFR	0.83 [0.8 to 0.86]	0.84 [0.81 to 0.87]	0.84 [0.82 to 0.87]	0.84 [0.81 to 0.86]	0.85 [0.82 to 0.88]
	CER/height^2^	−0.2 [−0.23 to −0.17]	−0.18 [−0.21 to −0.15]	−0.18 [−0.21 to −0.16]	−0.14 [−0.17 to −0.12]	−0.19 [−0.22 to −0.17]
*R* ^2^		0.65	0.66	0.67	0.66	0.68

*Note:* CER was scale to the square of height (CER/height^2^).

Abbreviations: *β* (95% CI), *β* coefficient (95% confidence interval).

**TABLE 4:** Performance of the 4 eGFRcr equations according to gender specific CER/Height^2^ tertile.

CER/Height^2^ tertile		Tertile 1	Tertile 2	Tertile 3
ioGFR (ml/min/1.73 m2)	Mean ± SD	47 ± 15	54 ± 14	57 ± 15
MDRD				
Correlation coefficient	CC (95% CI)	0.83 (0.81–0.86)	0.83 (0.8–0.85)	0.8 (0.77–0.83)
Median absolute bias (mL/min/1.73m^2^)	Median [Q25, Q75]	3.3 [−3, 10.2]	−1.5 [−6.7, 5.1]	−7.32 [−13.2, −1.5]
Percentage within 30% of ioGFR	% (95% CI)	78.5 (75.1–81.6)	91.8 (89.4–93.7)	83.9 (80.8–86.6)
Percentage within 10% of ioGFR	% (95% CI)	33.3 (29.7–37.2)	41.9 (38–45.8)	30.9 (27.3–34.6)
CKD‐EP_2009_				
Correlation coefficient	CC (95% CI)	0.85 (0.82–0.87)	0.82 (0.79–0.85)	0.8 (0.77–0.82)
eGFR‐mGFR (mL/min/1.73m^2^)	Median [Q25, Q75]	4.5 [−2.5, 12.5]	1.5 [−5.2, 9.3]	−4.24 [−10.3, 2.5]
Percentage within 30% of ioGFR	% (95% CI)	66.7 (62.8–70.3)	81.7 (78.4–84.5)	88.2 (85.4–90.5)
Percentage within 10% of ioGFR	% (95% CI)	24.8 (21.5–28.4)	33.6 (29.9–37.4)	40.2 (36.4–44.2)
CKD‐EPI_2021_				
Correlation coefficient	CC (95% CI)	0.85 (0.82–0.87)	0.83 (0.8–0.85)	0.8 (0.77–0.83)
eGFR‐mGFR (mL/min/1.73m^2^)	Median [Q25, Q75]	7.5 [0.1, 15.9]	4.1 [−2.4, 12.5]	−1.5 [−8.1, 5.2]
Percentage within 30% of ioGFR	% (95% CI)	66.7 (62.8–70.3)	81.7 (78.4–84.5)	88.2 (85.4–90.5)
Percentage within 10% of ioGFR	% (95% CI)	29.1 (25.6–32.8)	38.8 (35–42.7)	37.8 (34–41.7)
EKFC				
Correlation coefficient	CC (95% CI)	0.84 (0.81–0.86)	0.82 (0.79–0.84)	0.8 (0.77–0.83)
eGFR‐mGFR (mL/min/1.73m^2^)	Median [Q25, Q75]	3.5 [−3.3, 10.9]	1.1 [−5.3, 7.9]	−3.9 [−10, 2.5]
Percentage within 30% of ioGFR	% (95% CI)	76.5 (73–79.7)	87.2 (84.4–89.6)	89.5 (86.8–91.7)
Percentage within 10% of ioGFR	% (95% CI)	29.7 (26.2–33.5)	38.8 (35–42.7)	40.1 (36.2–44)
RFKS				
Correlation coefficient	CC (95% CI)	0.84 (0.82–0.86)	0.83 (0.8–0.85)	0.8 (0.77–0.83)
eGFR‐mGFR (mL/min/1.73m^2^)	Median [Q25, Q75]	5.4 [0.2, 10.9]	1.3 [−3.5, 6.4]	−3.4 [−9.5, 2.3]
Percentage within 30% of ioGFR	% (95% CI)	79.5 (76.1–82.5)	93.3 (91–95)	93.9 (91.7–95.6)
Percentage within 10% of ioGFR	% (95% CI)	34.5 (30.8–38.3)	50.7 (46.8–54.7)	44.7 (40.8–48.6)

Abbreviations: 95% CI, 95% confidence interval; ioGFR, GFR measured with iohexol.

### 3.4 Association Between eGFRcr, ioGFR and Mortality

After a mean follow‐up of 56 ± 34 months, 142 patients lost their allograft, 216 patients died (including 16 who died after returning to dialysis) and 86 patients were lost to follow‐up. In univariate analysis, eGFRcr was associated with mortality whatever the equation used. We also observed an association with mortality for ioGFR (HR 0.96, 95% CI [0.96–0.97], *p* < 0.001) and muscle mass (HR 0.78, 95% CI [0.73–0.82], *p* < 0.001; Table S1).

In multivariable analyzes unadjusted for CER, the association between eGFRcr and mortality was not significant, irrespective of the equation. However, the association between ioGFR and mortality remained significant (HR 0.987, 95% CI [0.972–0.995], *p* = 0.006). When we added CER in the models, the association between ioGFR and mortality decreased but remained significant (HR 0.987, 95% CI [0.976–0.999], *p* = 0.045). In contrast, the HR for mortality for all eGFRcr equations increased, leading to a significant association for MDRD and RFKS (MDRD: HR 0.990, 95% CI [0.981–0.999], *p* = 0.03; RFKS: HR 0.987, 95% CI [0.976–0.999], *p* = 0.03). Results are summarized in Table 5.

**TABLE 5:** Multivariate analysis of the association between GFR and mortality.

	Model without CER^a^			Model with CER^b^		
	Hazard ratio	95% CI	*p*‐value	HR	95%CI	*p‐*value
ioGFR	0.983	(0.972–0.995)	0.006	0.987	(0.976–0.999)	0.04
CER	—	—	—	0.811	(0.708–0.914)	< 0.001
MDRD	0.992	(0.983–1.001)	0.08	0.990	(0.981–0.999)	0.03
CER	—	—	—	0.782	(0.681–0.882)	< 0.001
CKDEPI_2009_	0.993	(0.984–1.002)	0.1	0.992	(0.983–1.000)	0.06
CER	—	—	—	0.783	(0.683–0.883)	< 0.001
CKDEPI_2021_	0.993	(0.985–1.002)	0.1	0.992	(0.984–1.000)	0.06
CER	—	—	—	0.783	(0.683–0.883)	< 0.001
EKFC	0.993	(0.984–1.003)	0.2	0.991	(0.982–1.001)	0.08
CER	—	—	—	0.784	(0.683–0.884)	< 0.001
RFKS	0.99	(0.979–1.001)	0.07	0.987	(0.976–0,999)	0.03
CER	—	—	—	0.782	(0.682–0.882)	< 0.001

^a^ Model adjusted for age, gender, systolic blood pressure, height, weight, tobacco use, coronary disease, diabetes, preemptive transplantation, donor age, type of donor (deceased or living), transplantation vintage, proteinuria.

^b^ Model adjusted for age, gender, systolic blood pressure, height, weight, tobacco use, coronary disease, diabetes, preemptive transplantation, donor age, type of donor (deceased or living), transplantation vintage and urinary creatinine excretion rate, and proteinuria.

Finally, we assessed the association of non‐GFR determinants of eGFRcr with mortality by adjusting the HR for mortality of eGFRcr for age, gender and ioGFR (Table 6). Strikingly, ioGFR adjustment inverted the relationship between eGFRcr and mortality, with high eGFRcr paradoxically predicting increased mortality. Collectively, these results indicate that muscle mass, as a non‐GFR determinant of eGFRcr, measurably confounds the association of eGFRcr with mortality.

**TABLE 6:** Analysis of the association of the non‐GFR determinant of eGFRcr with mortality.

	Adjusted for sex and age			+ ioGFR		
	Hazard ratio	95% CI	*p*‐value	HR	95% CI	*p*‐value
ioGFR	0.983	(0.972–0.995)	0.006	—	—	—
MDRD	0.992	(0.998–0.999)	0.05	1.02	(1.004–1.027)	0.009
ioGFR	—	—	—	0.962	(0.946–0.977)	< 0.001
CKDEPI_2021_	0.993	(0.998–1)	0.06	1.02	(1–1.000)	0.06
ioGFR	—	—	—	0.962	(0.946–0.977)	< 0.001
EKFC	0.993	(0.984–1.003)	0.08	1.02	(1.008–1.034)	0.002
ioGFR	—	—	—	0.958	(0.942–0.974)	< 0.0001
RFKS	0.99	(0.99–0.999)	0.03	1.02	(1.005–1.035)	0.01
ioGFR	—	—	—	0.96	(0.944–0.977)	< 0.0001

Abbreviations: 95% CI, 95% confidence interval; ioGFR, GFR measured with iohexol.

## 4 Discussion

This study clarifies the impact of muscle mass on the performance of eGFRcr equations in KTR. First, we observed that muscle mass substantially impacts eGFRcr for all the tested equations. Indeed, eGFRcr accuracy was reduced in the tertile of patients with the lowest muscle mass irrespective of the formula. The KTR specific RFKS equation tends to perform a little better than the other equations. However, a large fraction of our cohort contributed to the development of this equation, and a better performance is therefore expected. In spite of this, the RFKS equation is at least as affected by muscle mass as the other equations. These results suggest that a specific calibration of eGFRcr equations in KTR does not reduce the weight of muscle mass as a non‐GFR determinant of eGFRcr. This result is significant: among the well‐established non‐GFR determinants of plasma creatinine, muscle mass is the only one for which variation with the two main parameters used in eGFR equations (gender and age) is clearly established. Thus, minimizing the impact of muscle mass on eGFRcr is a desirable effect of an eGFRcr equation. With this perspective, our results suggest that the tested equations have overall similar behaviours, with the exception of EKFC, for which the contribution of muscle mass to eGFRcr was lower than for the other equations in the study population. This finding may be related to the design of the EKFC equation, which is notably based on the observed variation of plasma creatinine with age in a large European population. Yet, EKFC formula results were nonetheless affected by muscle mass, and overestimation of the kidney function of patients with sarcopenia remained an important concern.

Our results also highlight how unmeasured muscle mass confounds studies relying on eGFRcr to assess the association of allograft kidney function and mortality. ioGFR showed a stronger association with mortality than eGFRcr in multivariable analyses neglecting muscle mass. In contrast, ioGFR and eGFRcr showed essentially similar performance in predicting mortality when CER was included in the models. This effect has two determinants. First, taking into account CER increases the association of eGFRcr with mortality. Second, adjusting for CER also reduces the association of mGFR with mortality. We previously showed that a low GFR after KT was associated with reduced muscle mass [8]. Thus, our results indicate that part of the effect of ioGFR on survival is mediated through its effect on muscle mass.

The magnitude of the confounding effect of muscle mass on the association of eGFRcr with mortality is however low and likely not relevant for most clinical situations. Yet, this effect is to be taken into account in large epidemiological studies investigating the factors influencing KT outcomes. In contrast, the impact of muscle mass on eGFRcr bias is relevant to the clinic. Indeed, 25% of the patients in the lower tertile of CER/Height^2^ had a mGFR at least 10 mL/min/1.73m^2^ lower than predicted by most eGFR equations. Such an important difference may affect drug dosage adaptation as well as the overall assessment of patients.

The implementation of additional endogenous GFR markers that are less affected by muscle mass, such as Cystatin C, may increase the accuracy of eGFR formulae, especially in patients with a low muscle mass. Yet, other confounding factors affect plasma Cystatin C level, and studies assessing the benefit of implementing cystatin C in eGFR equations showed conflicting results in KTR [15, 23, 24].

Our study has limitations. Muscle mass was evaluated solely through CER, and we did not perform any functional evaluation of muscle function. Yet, CER has been shown to correlate with the hand‐grip test in KTR [25]. We also did not measure cystatin C or other potential GFR markers. Finally, this study was conducted in a single, specialized transplant centre, which may limit the generalizability of the findings to other settings with different patient profiles or clinical practices. However, the standardized follow‐up and consistent data collection within the centre support the internal consistency of the results. Granted, our study also has several strengths. We studied a large, well‐phenotyped cohort with a prolonged follow‐up. GFR was measured with a gold standard reference that gives a value independent of muscle mass. Creatinine measurements were performed prospectively with a stable enzymatic method traceable to IDMS throughout the study.

This study, which is the first to measure how muscle mass affects eGFRcr formula performance after KT, demonstrated a clinically relevant reduction in accuracy in patients with a low muscle mass. As these patients also display lower GFR and have a higher risk of death, this study highlights the importance of evaluating muscle mass in KTR. Our study further demonstrates that the weight of muscle mass as a non‐determinant of eGFRcr reduces the ability of these estimators to capture the mortality burden linked to reduced kidney allograft function. A systematic assessment of muscle mass in KTR may allow the identification of a population of frail patients in whom eGFRcr underestimates kidney function impairment.

### Ackowledgements

The authors thank: Vincent Benoit for setting up the physiology department data base, Marie‐Louise Sileber, Audrey Tiquant, Khalil El Karoui, Marie Courbebaisse, Dominique Eladari, Ghania Daoud and the laboratory staff of the physiology department of Necker hospital for their help in collecting data, Adel Abderrahmane for his help with the DIVAT database and Thao Nguyen for her advice regarding creatinine measurement technique.

## Disclosure

The authors have nothing to report.

## Supporting information


**Figure S1.** Correlation matrix between ioGFR and covariates included in the multivariable Cox regression model.
**Table S1.** Univariate association with mortality.

## Data Availability

The datasets generated during and/or analysed during the current study are available from the corresponding author on reasonable request.
