# Supplementary material for: Impact of Muscle Mass on the Performance of Creatinine‐Based eGFR Equations and Mortality Risk Assessment After Kidney Transplantation
Source: J Cachexia Sarcopenia Muscle. 2025 Sep 29;16(5):e70032. doi: 10.1002/jcsm.70032 (PMC12478446; doi:10.1002/jcsm.70032)
Supplement: Supplementary file 1 — Figure S1. Correlation matrix between ioGFR and covariates included in the multivariable Cox regression model. Table S1. Univariate association with mortality. [file JCSM-16-e70032-s001.docx]

Impact of muscle mass on the performance of creatinine-based eGFR equations and mortality risk assessment after kidney transplantation

François Gaillard^1,2^, Melissa Ould Rabah^3,4^, Olivier Aubert^4,5,6^, Nicolas Garcelon^7^, Antoine Neuraz^8^, Christophe Legendre^4,5,6^, Dany Anglicheau^4,5,6^, Dominique Prié^3,4,6^, and Frank Bienaimé^3,4,6^

**Suplemmentary figure 1**: Correlation matrix between ioGFR and covariates included in the multivariable Cox regression model.

The figure displays Pearson correlation coefficients between measured glomerular filtration rate (iohexol clearance, ioGFR) and key clinical covariates: age, gender, systolic blood pressure, height, weight, tobacco use, coronary artery disease, diabetes, preemptive transplantation, donor age, type of donor, time on dialysis before transplantation (vintage), creatinine excretion rate and proteinuria. The strength and direction of correlations are indicated by color intensity and numeric values. ioGFR was not strongly correlated with other covariates.

**Supplementary table 1: Univariate association with mortality.**

BMI: Body mass index, CER: Creatinine excretion rate.

|  | **HR** | **95%CI** | **P-value** |
| --- | --- | --- | --- |
| Age (years) | 1.1 | (1.1-1.1) | <0.001 |
| Male gender | 1.2 | (0.93-1.6) | 0.16 |
| Current smoker | 0.94 | (0.63-1.4) | 0.77 |
| Coronary disease | 3.8 | (2.8-5) | <0.001 |
| Diabetes | 2.8 | (2.1-3.7) | <0.001 |
| BMI (kg/m^2^) | 1 | (1-1.1) | 0.044 |
| Systolic blood pressure (mmHg) | 1 | (1-1) | <0.001 |
| Log-Proteinuria / Creatininuria (mg/mmol) | 2.6 | (1.9-3.5) | <0.001 |
| Preemptive transplantation | 0.33 | (0.19-0.57) | <0.001 |
| Donor age (years) | 1 | (1-1.1) | <0.001 |
| Living donor | 0.3 | (0.19-0.46) | <0.001 |
| CER (µmol/min) | 0.78 | (0.73-0.82) | <0.001 |
| ioGFR (mL/min/1.73m^2^) | 0.96 | (0.96-0.97) | <0.001 |
| eGFR-MDRD (mL/min/1.73m^2^) | 0.99 | (0.98-0.99) | <0.001 |
| eGFR-CKDEPI_2009_ (mL/min/1.73m^2^) | 0.98 | (0.97-0.99) | <0.001 |
| eGFR-CKDEPI_2021_ (mL/min/1.73m^2^) | 0.98 | (0.98-0.99) | <0.001 |
| eGFR-EKFC (mL/min/1.73m^2^) | 0.98 | (0.97-0.99) | <0.001 |
| eGFR-RFKTS (mL/min/1.73m^2^) | 0.97 | (0.97-0.98) | <0.001 |
